# Retrospective cohort evaluation of renal involvement in non-HIV castleman disease patients from a single academic center in Beijing, China

**DOI:** 10.1007/s00277-026-06739-1

**Published:** 2026-01-15

**Authors:** Hongtao Ling, Lihong Wang, Wei Wang, Xiaoying Yang, Wenqiong Wang, Shuanglian Xie, Yiming Zhao, Shujing Guo, Weiwei Xie, Zhizhen Lai, Huihui Liu, Xiaodi Yang, Xiaojuan Yu, Yujun Dong

**Affiliations:** 1 Department of Hematology, Peking University First Hospital, No. 8 Xishiku St., Xicheng Distr., 100034 Beijing People’s Republic of China; 2 Department of Pathology, Peking University First Hospital, No.8 Xishiku St. Xicheng Distr., Beijing, People’s Republic of China; 3 Department of Nephrology, Peking University First Hospital, No.8 Xishiku St. Xicheng Distr., Beijing, 100034 People’s Republic of China

**Keywords:** Castleman disease, Renal involvement, Clinical characteristics, Renal pathology

## Abstract

Castleman disease (CD), a rare and clinically heterogeneous condition, frequently involves renal impairment, though this relationship remains poorly characterized. This large cohort study of 183 patients (116 unicentric [UCD], 67 multicentric [MCD]) investigated renal involvement (RI). RI occurred in 6.03% (7/116) of UCD and 55.22% (37/67) of MCD patients. In UCD-RI, 4 underwent renal biopsy, revealing varied pathological results, and 1 underwent total left nephroureterectomy. In MCD-RI, common manifestations included edema, nephrotic syndrome, and acute renal failure. Thrombotic microangiopathy (TMA) was the most frequent renal pathology (9/19 biopsies). Acute renal failure often responded well to treatment, with 60% (9/15) achieving complete recovery. The myeloma-like treatment regimen demonstrated superior efficacy compared to the lymphoma-like regimen and immunomodulatory therapy (*P* = 0.039). The 5-year renal survival rate in the MCD-RI group was 88.9%, not significantly different from UCD-RI (*P* = 0.45). Furthermore, the 5-year overall survival in the CD-RI group was 81.9%, showing no statistically significant difference from CD patients without renal involvement (*P* = 0.11). This study confirms that RI is more common in MCD, with TMA as a key pathological feature, and demonstrates that renal involvement does not negatively impact overall survival.

**Supplementary Information:**

The online version contains supplementary material available at 10.1007/s00277-026-06739-1.

##  Introduction

Castleman disease (CD) is a rare lymphoproliferative disorder first described in 1956 [1]. Its position within the disease spectrum remains unclear, spanning hematology, oncology, rheumatology, and virology [2]. Clinically, CD is classified into unicentric (UCD) and multicentric (MCD) forms. As early as 2012, our hospital reported an initial cohort of CD patients with renal involvement, identifying thrombotic microangiopathy (TMA) as a common pathological feature [3]. This study describes a larger cohort from the same center in North China, focusing on renal involvement and prognosis in 183 CD patients. It is now widely accepted internationally that UCD and MCD are distinct entities, both in terms of clinical course and underlying pathogenesis [4, 5]. Therefore, we have categorized renal involvement in Castleman disease into UCD and MCD groups, and will analyze and compare various clinical indicators and prognosis within each group.

##  Methods

### Study design and participants

This real-world, retrospective study enrolled patients diagnosed with CD at Peking University First Hospital between April 1, 2007, and April 30, 2025. The study was approved by the hospital’s Ethics Committee, and all study procedures followed the ethical standards of the Declaration of Helsinki. Patients were rigorously screened according to the 2025 Chinese Castleman Disease Network (CCDN) guidelines [6], excluding other diseases causing Castleman-like lymphadenopathy. All cases were diagnosed by experienced pathologists and confirmed based on their imaging studies (including CT, PET/CT) to determine the areas of lymph node involvement. Demographic, clinical, and laboratory data were collected retrospectively. We followed up with all patients via telephone and text message until July 2025. For lost-to-follow-up cases, we used the last visit date obtained from the Peking University First Hospital outpatient and inpatient medical record system as a substitute for the follow-up date.

### Measurements and definitions

Castleman disease with renal involvement (CD-RI) was defined by the presence of at least one of the following: (1) hematuria, > 3 red blood cells per high-power field (HPF); (2) proteinuria, urinary protein > 150 mg/day; (3) renal dysfunction, estimated glomerular filtration rate (eGFR) < 60 ml/min/1.73 m²; or (4) No previous history of kidney disease. Other definitions in this study included: anemia defined as hemoglobin < 100 g/L, thrombocytopenia defined as platelets < 100 × 10⁹/L [6], elevated erythrocyte sedimentation rate (ESR) defined as > 15 mm/h for males and > 20 mm/h for females, elevated serum creatinine defined as > 133 µmol/L, hypoalbuminemia defined as plasma albumin < 35 g/L, elevated C-reactive protein (CRP) defined as > 10 mg/L, elevated serum IgG defined as > 17 g/L, elevated interleukin-6 (IL-6) defined as > 6.4 pg/ml, and elevated vascular endothelial growth factor (VEGF) defined as > 142 pg/ml. Overall survival (OS) was defined as the time interval from the date of CD diagnosis to death from any cause or the last follow-up date. Renal survival rate was defined as the proportion of patients who maintained survival without dependence on hemodialysis or peritoneal dialysis.

### Renal pathology

Patients with CD underwent renal biopsy if they met one or more of the following criteria: rising serum creatinine, proteinuria ≥ 0.5 g/24 hr, or complete/partial Fanconi syndrome [7]. Renal biopsy specimens were examined by direct immunofluorescence, light microscopy (LM), and electron microscopy (EM) techniques. Specimens were fixed in 4.5% buffered formaldehyde for LM, and part of the sample was fixed in 2.5% paraformaldehyde for EM. Consecutive serial 3-µm sections were used for histological staining. The following staining techniques were used: hematoxylin and eosin, periodic acid–Schiff, silver methenamine, and Masson’s trichrome. EM was performed according to standard procedures. After being embedded in epon, ultrathin sections were mounted on metal grids and stained with uranyl acetate before being viewed in a transmission electron microscope (JEM-1230; JEOL, Tokyo, Japan). Amyloidosis was diagnosed with the aid of Congo red staining. The pathological diagnoses of all renal biopsies were reviewed and confirmed by two experienced pathologists.

### Statistical analysis

Data were analyzed using SPSS 26.0 (IBM, Armonk, NY, USA) and R 4.5.1 (R Foundation for Statistical Computing, Vienna, Austria). In descriptive statistics, categorical variables were presented as frequencies (percentages), and continuous variables as medians (interquartile range, IQR); percentages were calculated after excluding missing values. Group comparisons employed stratified analysis methods: unordered categorical variables used Fisher’s exact test when cell expected frequencies were < 5, and Pearson’s chi-square test when ≥ 5; ordered categorical variables used the Wilcoxon signed-rank test; continuous variables, after Shapiro normality test (*p* > 0.05 for normal distribution), were analyzed using independent samples t-test for normally distributed data and the Mann-Whitney U test (for two groups) for non-normally distributed data. In survival analysis, Kaplan-Meier method was used to construct survival curves, and differences between groups were assessed by the Log-rank test. A P-value < 0.05 was considered statistically significant.

## Results

A total of 233 patients were initially suspected of having Castleman disease. Most patients with a single lesion underwent surgical resection of the affected area, and one patient underwent needle biopsy. For patients with multiple enlarged lymph nodes, we performed a biopsy on the largest superficial lymph node. For patients without superficial lymphadenopathy, we conducted needle biopsies on larger lymph nodes located in safe anatomical sites. Some patients with unsatisfactory biopsy results underwent subsequent surgical excision for histological examination. Twelve patients either refused surgical resection or could not be pathologically diagnosed with Castleman disease were excluded from this cohort.

**Fig. 1:**
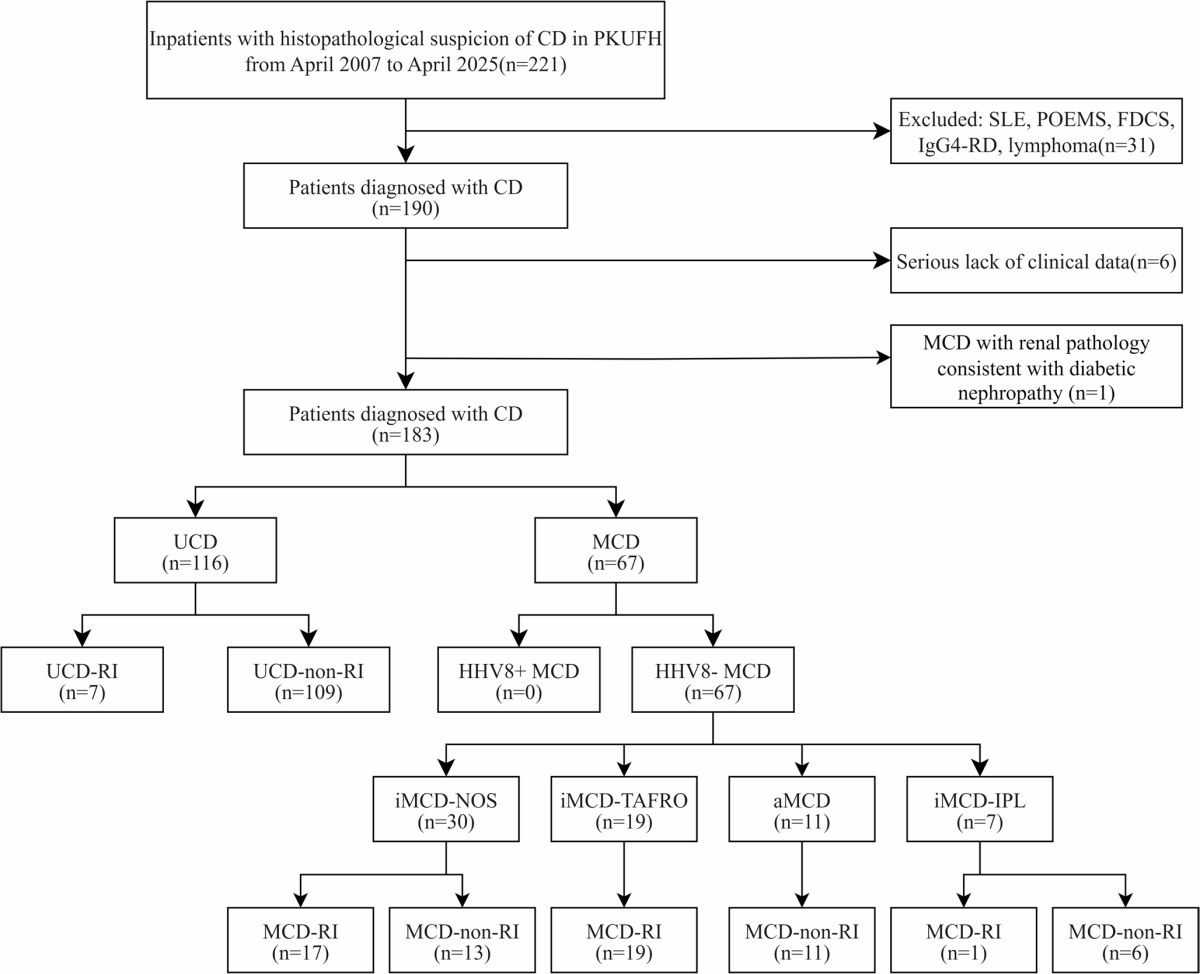
Patient selection and classification flowchart

ANA, ENA antibodies, and serum/urine immunofixation electrophoresis were routinely ordered for those patients with suspicious Castleman disease. Among the 221 patients initially screened with Castleman disease-like features, 31 were excluded due to other diseases (systemic lupus erythematosus, POEMS syndrome, follicular dendritic cell sarcoma, IgG4 related disease, lymphoma), and an additional 7 were excluded due to missing data or the presence of comorbid diabetic nephropathy. Ultimately, the cohort included 183 patients diagnosed with Castleman disease, categorized into unicentric Castleman disease (UCD, *n* = 116) and multicentric Castleman disease (MCD, *n* = 67). According to the newly released CDCN 2025 guidelines [6], multicentric Castleman disease was further classified into asymptomatic MCD (aMCD, *n* = 11), idiopathic MCD–not otherwise specified (iMCD-NOS, *n* = 30), idiopathic MCD–idiopathic plasmacytic lymphadenopathy (iMCD-IPL, *n* = 7), and idiopathic MCD–thrombocytopenia, anasarca, fever, reticulin fibrosis/renal dysfuction, and organomegaly (iMCD-TAFRO, *n* = 19). Either LANA1 staining or HHV8-DNA testing by Real-time PCR or both had been done for all of the MCD patient in this cohort. Neither of the HHV‑8 LANA-1 staining on pathological tissue nor HHV8 DNA real time PCR was positive (Fig. 1).

Based on the criteria for Castleman disease-related renal injury, 7 patients in the UCD group were identified as having renal involvement (UCD-RI). All iMCD-TAFRO patients exhibited renal involvement, while none of the aMCD patients did. Only 1 patient (14.3%) in the iMCD-IPL group showed renal involvement, and 17 patients (56.7%) in the iMCD-NOS group had renal involvement. In total, there were 37 patients with multicentric Castleman disease and renal involvement (MCD-RI).

### UCD-RI

Among the 116 UCD patients, 51 (43.9%) had concurrent paraneoplastic pemphigus (PNP), and 15 (12.9%) had bronchiolitis obliterans (BO). Among the 7 UCD-RI patients, 3 presented initially with symptoms such as edema and proteinuria and went directly to the Nephrology department, 1 patient presented to Respiratory Medicine, and 3 patients went directly to Surgery department. One of these 3 surgical patients had no clinical symptoms and reported solely with the chief complaint of “detected left-sided hydronephrosis.” The median age of the 7 patients was 34 years (range: 25–69), with a male-to-female ratio of 3:4. Regarding pathological type, 5 patients (71.4%) were diagnosed with the hyaline vascular (HV) type, and 2 (28.6%) with the plasma cell (PC) type. There were no statistically significant differences between the renal involvement and non-renal involvement groups in terms of gender ratio, age at diagnosis, time from onset to diagnosis, or pathological type. One male patient had concurrent paraneoplastic pemphigus (PNP), while the remaining patients had no comorbidities.

Among the 7 UCD-RI patients, 3 (42.9%) presented with at least one of the following: fever, fatigue, decreased appetite, and/or weight loss. Two patients (28.6%) had ascites and/or pleural effusion; no patients had pericardial effusion, hepatomegaly, or splenomegaly. Regarding renal manifestations, 5 patients (71.4%) presented clinically with hematuria, 3 (42.9%) with nephrotic syndrome, 1 (14.3%) with acute renal failure, and 1 (14.3%) with hydronephrosis of the left kidney due to compression by a mass in the left renal pelvis and ureter.

Presumably due to the small number of patients in the UCD-RI group, there were no statistically significant differences in anemia, thrombocytopenia, or serum creatinine. For elevated CRP, hsCRP, ESR, IL-6, and IgG, there was considerable missing data, most severely for VEGF, where no patient in the UCD-RI group had VEGF tested, and only 3 in the UCD-non-RI group did. In these aspects, there were no statistically significant differences between the two groups. (Table 1)

**Table 1:** Laboratory parameters of UCD-RI patients

	UCD-RI (*n* = 7)	UCD-non-RI (*n* = 109)	*P* value
Anemia, n(%)	1/7 (14.3%)	6/109 (5.5%)	0.361
Thrombocytopenia, n(%)	1/7 (14.3%)	1/109 (0.9%)	0.118
Hypoalbuminemia, n(%)	4/7 (57.1%)	31/106 (29.2%)	0.2
Scr, µmol/L	76.43(66–89.18.18)	66.35(58.4–79.75.4.75)	0.077
Elevated Scr	0/7	0/108	
Elevated CRP, n(%)	2/3 (66.7%)	9/16 (56.3%)	1
Elevated hsCRP, n(%)	2/4 (50%)	15/38 (39.5%)	1
Elevated ESR, n(%)	3/5 (60%)	21/42 (50%)	1
Elevated IL-6, n(%)	1/3 (33.3%)	4/7 (57.1%)	1
Elevated VEGF, n(%)	0/0	3/3 (100%)	
Elevated IgG, n(%)	1/4 (25%)	4/22 (18.2%)	1

**Fig. 2:**
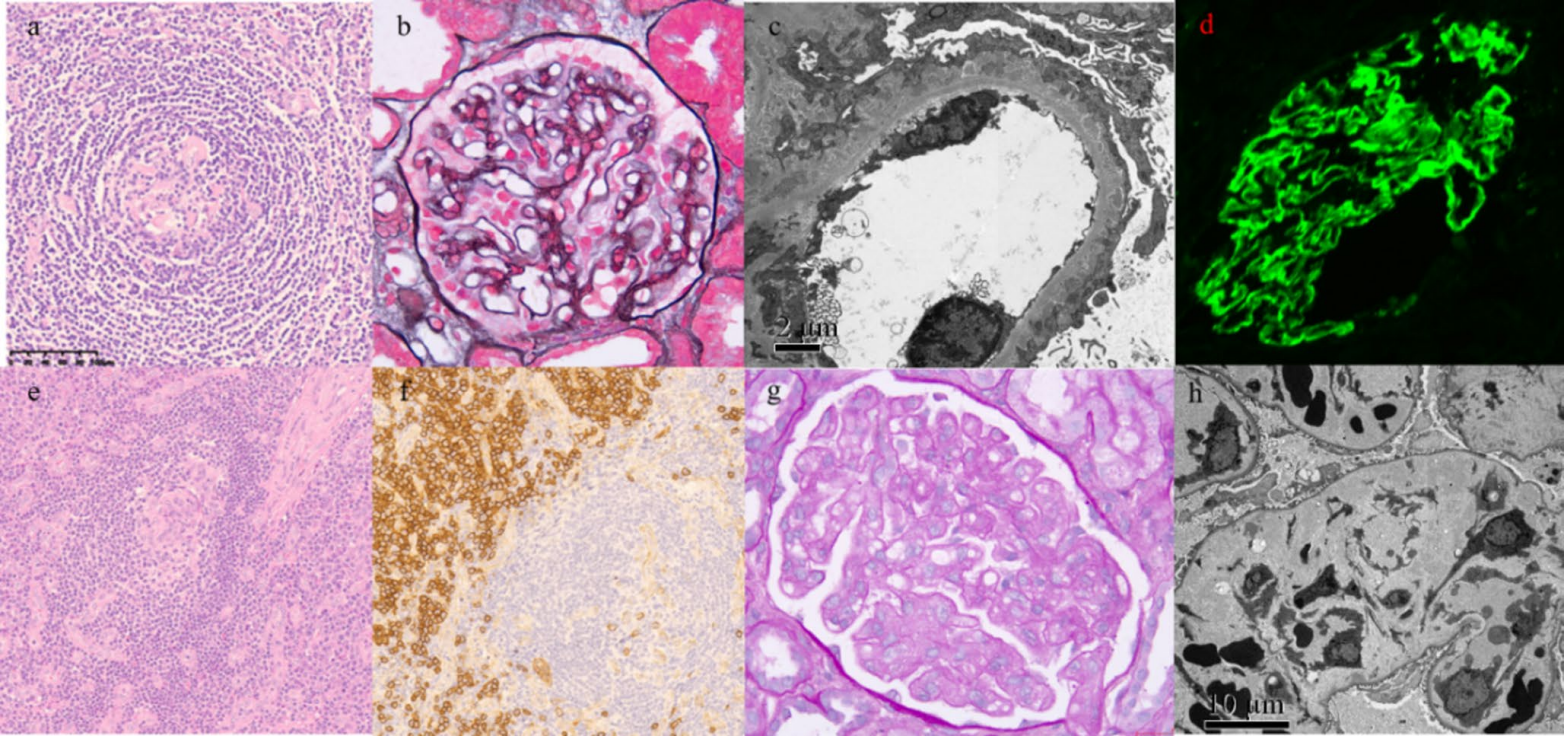
Presents lymph node and renal pathology from two patients. a-d (Patient 1): Hyaline vascular (HV) lymph node and membranous nephropathy. (**a**) Hematoxylin and eosin (HE) ×20: Onion-skin appearance. (**b**) PASM + Masson ×400: Subepithelial immune deposits. (**c**) Electron microscopy (EM) & (**d**) immunofluorescence electron microscopy: Subepithelial/intramembranous electron-dense deposits. e-h (Patient 2): Mixed-type lymph node and thrombotic microangiopathy (TMA). (**e**) HE ×20: Onion-skin appearance. (**f**) CD138 × 20: Sheets of plasma cells. (**g**) PASM + Masson ×400: Segmental double contours. (**h**) EM: No electron-dense deposits

Four of the 7 patients underwent renal biopsy, revealing varied pathologies: TMA, focal segmental glomerulosclerosis (perihilar variant), membranous nephropathy, and ischemic kidney injury. (Fig. 2)

### MCD-RI

There were 37 MCD-RI cases. Among them, 22 patients presented initially with urinary system symptoms and went directly to Nephrology, 11 patients presented to Hematology, 2 to Gastroenterology, 1 to Respiratory Medicine, and 1 to Infectious Diseases. Compared to MCD-non-RI patients, there were no significant differences in gender ratio (59.5% vs. 46.7%, *P* = 0.296) or median age at diagnosis (52 years vs. 45 years, *P* = 0.233). The proportion of the mixed (Mix) pathological type was significantly higher in the MCD-RI group (43.2%) than in the MCD-non-RI group (13.3%) (*P* = 0.029). Regarding clinical subtypes, all iMCD-TAFRO cases exhibited renal involvement and were classified in the MCD-RI group, while all asymptomatic MCD (aMCD) cases were classified in the MCD-non-RI group; the difference between the two groups was statistically significant (*P* < 0.001). (Supplement Table 1)

There were no significant statistical differences between MCD-RI and MCD-non-RI in the prevalence of fever (*P* = 0.176), night sweats (*P* = 0.684), or weight loss (*P* = 0.867). The proportions of patients with fatigue (73%) and decreased appetite (64.9%) were significantly higher in the MCD-RI group than in the MCD-non-RI group, with statistically significant differences (*P* < 0.001). The proportions of patients with pleural effusion (56.8%) and ascites (64.9%) in the MCD-RI group were higher than those in the MCD-non-RI group (pleural effusion 6.67%, ascites 3.33%), with statistically significant differences (*P* < 0.001). There were no significant differences between the two groups in hepatomegaly (*P* = 0.281), splenomegaly (*P* = 0.090), or rash (*P* = 0.650). (Supplement Table 2)

Analysis of urinary symptom patterns (Supplement Fig. 1) revealed that isolated edema and the combination of edema with hematuria were the most common patterns (9/37 each). No cases presented with isolated oliguria. Four patients in the MCD-RI group had no specific urinary symptoms and were found to have elevated serum creatinine only during physical examination; these are not shown in the figure.

The incidence rates of anemia, thrombocytopenia, and hypoalbuminemia were significantly higher in MCD-RI patients than in those without renal involvement (*P* < 0.05). For elevated CRP, ESR, IL-6, VEGF, and IgG, there was considerable missing data. A significant difference was observed only for elevated ESR (*P* = 0.016); there were no other statistically significant differences between the CD-RI and CD-non-RI groups for the remaining parameters. Given the unique nature of iMCD-TAFRO onset, after excluding the iMCD-TAFRO subgroup from the MCD-RI group and then comparing the MCD-RI group with the iMCD-non-RI group, likely due to the limited sample size, only decreased albumin, elevated creatinine, and abnormal urinalysis showed statistically significant differences (*P* = 0.001), while no significant differences were observed in the remaining parameters (Table 2).

**Table 2:** Laboratory parameters of MCD-RI patients

	MCD-RI (*n* = 37)	MCD-non-RI (*n* = 30)	*P* value^a^	MCD-RI without TAFRO (*n* = 18)	*P* value^b^
Anemia, n(%)	22/37 (59.5%)	8/30 (26.7%)	0.013	9/18(50%)	0.102
Thrombocytopenia, n(%)	11/37 (29.7%)	1/30 (3.3%)	0.005	1/18(5.6%)	1
Hypoalbuminemia, n(%)	34/37 (91.9%)	14/30 (46.7%)	<0.001	17/18(94.4%)	0.001
Scr, µmol/L	146.06(107.95–246.35.95.35)	64.85(55.78–80.25)	<0.001	142.7(101.73–228)	<0.001
Scr>133umol/L	23/37 (62.2%)	0	<0.001	11/18(61.1%)	<0.001
eGFR<60, n(%)	30/37(81.1%)	0	<0.001	13/18(72.2%)	<0.001
Positive occult blood in urine, n(%)	21/37(56.8%)	0	<0.001	11/18(61.1%)	<0.001
Positive urine protein, n(%)	33/37(89.2%)	0	<0.001	16/18(88.9%)	<0.001
Elevated CRP, n(%)	16/23 (69.6%)	7/15 (46.7%)	0.158	4/9(44.4%)	1
Elevated hsCRP, n(%)	13/20 (65%)	9/18 (50%)	0.35	6/9(66.7%)	0.683
Elevated ESR, n(%)	29/34 (85.3%)	13/23 (56.5%)	0.016	14/18(77.8%)	0.154
Elevated IL-6, n(%)	11/14 (78.6%)	8/11 (72.7%)	1	7/8(87.5%)	0.603
Elevated VEGF, n(%)	8/13 (61.5%)	1/5 (20%)	0.294	3/4(75%)	0.206
Elevated IgG, n(%)	15/33 (45.5%)	11/23 (47.8%)	0.861	10/17(58.8%)	0.491

^a^Statistical comparison between MCD-RI and MCD-non-RI; ^b^Statistical comparison between MCD-non-RI and MCD-RI without the TAFRO subgroup

Among the 37 MCD-RI patients, 14 had nephrotic syndrome, 14 had acute renal failure, 13 had chronic renal insufficiency, and 1 had rapidly progressive glomerulonephritis (RPGN). Seven patients required emergent renal replacement therapy, and 3 progressed to end-stage renal disease (ESRD). At initial treatment, 15 patients had an eGFR < 30 ml/min/1.73 m², 15 had an eGFR between 30 and 60 ml/min/1.73 m², and the remaining 7 had an eGFR greater than 60 ml/min/1.73 m².

Among the 19 iMCD-TAFRO patients, 8 (42.1%) underwent renal biopsy, with 6 (75%) showing renal pathology consistent with thrombotic microangiopathy (TMA), featuring endothelial swelling, widening of the subendothelial space with double contours, or subendothelial accumulation of protein and debris. (Fig. 2) One patient had pathology consistent with subacute tubulointerstitial nephropathy, and one patient tested positive for anti-GBM antibodies, with renal biopsy pathology revealing membranous nephropathy combined with type 2 crescentic glomerulonephritis. Among the 17 iMCD-NOS patients with renal involvement, 10 (58.8%) underwent renal biopsy. The renal pathology findings included TMA in 3 (30%) patients, IgA nephropathy in 2 patients, chronic tubulointerstitial nephritis (CTIN) in 1 patient, and membranous nephropathy in 1 patient. The remaining 3 patients were suspected to have IgG4-related disease (IgG4-RD) based on renal pathological examination. Both patients who tested positive for anti-neutrophil cytoplasmic antibodies (ANCA) underwent renal biopsy: one showed mild mesangial proliferative IgA nephropathy with acute renal failure, and the other showed TMA without acute renal failure. The one iMCD-IPL patient with renal involvement underwent renal biopsy, with pathology revealing focal IgA nephropathy with acute tubulointerstitial nephritis. No statistically significant difference was observed in the presence of thrombotic microangiopathy (TMA) on renal pathology between the iMCD-TAFRO and iMCD-NOS groups (*P* = 0.153). In the cohort, one patient with iMCD-TAFRO showed positive Congo red staining on both lymph node and abdominal wall fat biopsies.

### Clinical treatment and follow-up

All 7 patients in the UCD-RI group underwent surgical treatment. Among them, three patients were lost of follow-up, 3 of the rest 4 patients received glucocorticoid based therapy. All of the 4 patients achieved complete renal remission during follow-up.(Supp. Figure 2) Among the 19 iMCD-TAFRO patients, 10 received lymphoma-like regimens, 6 received myeloma-like regimens, 1 received steroid therapy alone, the other 2 patients lacked data on the initial treatment for renal injury and its response. Complete renal remission was achieved in 13 patients (68.4%). 4 patients did not response to treatment, including the two cases with positive anti-GBM antibodies progressed to end-stage renal disease. (Supp. Figure 2) Among the 17 iMCD-NOS patients with renal involvement, 7 received lymphoma-like regimens, 2 received steroid alone, 1 was treated with siltuximab combined with PD (pomalidomide and dexamethasone), 1 received steroids combined with azathioprine, the other 6 patients lacked data on renal injury treatment, response, and follow-up. Complete renal remission was achieved in 5 patients (29.4%),. Two patients achieved partial renal remission. The complete remission rate was significantly higher in iMCD-TAFRO patients than in iMCD-NOS patients (*P* = 0.039). A statistically significant difference in remission rates was observed among these three treatment groups (*P* = 0.039).

**Fig. 3:**
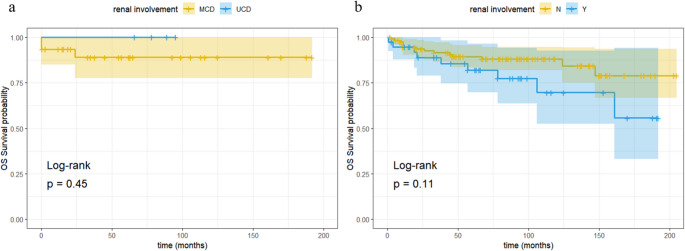
Subgroup overall survival analysis. **A** Renal survival analysis based on clinical subtypes; **B** overall survival analysis of CD patients based on with/without renal involvement

In summary, among the 44 patients with renal involvement, 14 were lost to follow-up. The remaining 30 patients had a median follow-up time of 61 months (IQR: 20.5–107.75 months). Nine patients (9/30, 30.0%) with renal involvement and 14 patients (14/92, 15.2%) without renal involvement died during the follow-up period. One patient (1/9, 11.1%) in the renal involvement group died from renal failure; other causes of death included pancreatic cancer, infection, respiratory failure, etc. The 5-year renal survival rate in the MCD-RI group was 88.9% (Fig. 3a), showing no statistically significant difference compared to the UCD-RI group (*P* = 0.45). The 5-year overall survival (OS) rate in the CD-RI group was 81.9% (Fig. 3B). The median OS was 145.68 months (95% CI, 120.38–170.97), which was not statistically significantly different from the CD-non-RI group (*P* = 0.11).

## Discussion

In this study, we characterized renal involvement in 183 Chinese patients with CD. Among them, 44 patients (24.0%) exhibited renal manifestations, including 7 with UCD and 37 with MCD, all of whom had been ruled out for common renal diseases (e.g., hypertension, diabetes, etc.). The most frequent clinical presentation was edema, while acute renal failure, nephrotic syndrome, and chronic renal insufficiency represented the predominant renal involvement patterns. Thrombotic microangiopathy-like lesions emerged as the most common pathological feature. We observed that the proportion of patients with thrombotic microangiopathy (TMA) on renal pathology was higher in the iMCD-TAFRO group than in the iMCD-NOS group. This difference did not, however, reach statistical significance, likely due to limited sample size. Most patients recovered from acute renal failure, particularly those in the iMCD-TAFRO group. In this cohort of patients, the myeloma-like regimen were found to have superior efficacy compared to both the lymphoma-like regimen and immunomodulatory therapy (*P* = 0.039). Given the limited numbers of each sub-group and the heterogenicity of the treatment regimens, further multi-centric prospective clinical trial is warranted to confirmed this result. Notably, renal involvement did not affect overall survival during follow-up.

It was previously believed that unicentric Castleman disease (UCD) rarely involved organ damage, except for potentially presenting with MCD-like systemic inflammatory reactions. This study included seven UCD patients with renal impairment, none of whom exhibited systemic inflammatory reactions. Their inflammatory markers—including ESR, CRP, hsCRP, and IL-6—showed no statistically significant differences compared to the UCD-non-RI group. Renal biopsy was performed in four of these patients, and the pathological findings varied considerably. One particularly notable case was asymptomatic and was only found to have left hydronephrosis caused by compression from a left ureteral mass on CT imaging, which is consistent with previous case reports of renal sinus Castleman disease [8]. Unlike those reports, however, pathological analysis in our case confirmed actual involvement of renal tissue, a feature shared with another reported case of renal pelvic Castleman disease [9]. Due to the rarity of Castleman disease and its atypical renal manifestations, the possibility of underdiagnosis or misdiagnosis remains substantial. Our previous studies have indicated that PNP significantly impacts the survival of patients with unicentric Castleman disease (UCD) [10, 11]. In this study, one of the UCD patients with renal impairment (UCD-RI) was also diagnosed with PNP and ultimately died from a pulmonary infection. This finding, to some extent, supports the conclusion drawn from our earlier research.

In recent years, it has become increasingly clear that even HHV-8-negative MCD is not a homogeneous entity [12]. Renal involvement has been reported in 25%–54% of CD cases, which is consistent with the findings of our study (24.0%). Small vessel damage characterized by thrombotic microangiopathy (TMA) is a typical feature of renal involvement in HIV-negative MCD [3, 13]. In this study, the MCD-RI group exhibited a higher prevalence of anemia, hypoalbuminemia, and elevated ESR compared to the MCD-non-RI group, which may be attributed to renal anemia and proteinuria. The higher incidence of thrombocytopenia is likely related to the inclusion of TAFRO subtype patients, all of whom presented with renal involvement. After excluding the TAFRO subgroup, only hypoalbuminemia showed a statistically significant difference between the MCD-non-RI and MCD-RI groups. This observation, however, may be at least partially related to the limited sample size. Notably, most patients in both the MCD-RI and MCD-non-RI groups showed varying degrees of elevated IL-6, suggesting a correlation between MCD pathogenesis and IL-6, consistent with previous studies [14, 15]. The proportion of elevated VEGF levels was higher in the MCD-RI group (61.5%) than in the MCD-non-RI group (20%), implying a potential association between renal involvement and VEGF, though the difference was not statistically significant due to the small sample size. Flahault et al. linked renal TMA manifestations to preeclampsia (related to soluble Flt1, which blocks VEGF signaling) and renal lesions during anti-VEGF antibody therapy [16]. El Karoui et al. observed reduced glomerular VEGF expression only in some patients with renal TMA and found that loss of glomerular VEGF expression was associated with elevated plasma CRP levels [13]. Renal TMA with endothelial swelling has also been described in patients treated with VEGF antibodies or sirolimus. Decreased glomerular VEGF leads to loss of fenestrations essential for glomerular filtration barrier permeability, resulting in microvascular damage and TMA [17]. The study by Sun et al. confirmed that plasma VEGF levels were significantly elevated in CD patients with TMA compared to those without TMA, suggesting that elevated plasma VEGF may be associated with TMA rather than CD itself [18]. In our cohort, two patients tested positive for anti‑GBM antibodies. The potential causal relationship between iMCD (or the TAFRO subtype) and anti‑GBM disease has been possibly linked to the production of antibodies reactive to the α3 chain of type IV collagen (α3(IV)NC1) by polyclonal plasma cells [19]. Similarly, we observed ANCA‑positive patients in the cohort. Thus iMCD might contribute to immunity disequilibrium and the production of auto-antibodies indirectly, which remains to be further validated.

Our cohort included three cases with renal pathology indicative of IgG4-related disease. However, these patients neither presented with eosinophilia nor demonstrated multi-system involvement. At least one of the clinical or pathological exclusion criteria is identified [20, 21, 22]. Consequently, a definitive diagnosis of IgG4-related disease could not be made. After discussion thoroughly with pathologists and rheumatologists, a consensus had been reached among our team that these three cases should be diagnosed as iMCDs, albeit their kidney lesions fulfilled the diagnostic criteria of IgG4-RD.

Regarding renal involvement and prognosis, although no statistically significant difference was observed between the CD-RI group and the CD-non-RI group (*P* = 0.11), this may be attributed to the relatively high number of lost-to-follow-up cases (33 patients) and an insufficient overall sample size, necessitating further expansion of the cohort for validation. Additionally, this study specifically introduced the concept of renal survival and found no significant difference in renal survival between the UCD and MCD subgroups (*P* = 0.45). Further comprehensive analysis incorporating other factors is required to draw more definitive conclusions. Only one patient presented with acute renal failure at onset, received treatment with corticosteroids combined with cyclophosphamide, and ultimately died due to renal failure. This finding contrasts with the conclusions reported by Zhang et al. in 2016 [5]. The discrepancy may be attributed to the following factors: (i) Differences in inclusion criteria—our study classified patients with proteinuria into the CD-RI group even when renal function was normal; (ii) Recent advances in treatment modalities may have contributed to improved patient outcomes. Although the concurrences of kidney injury and CD were reported and analyzed in this cohort, the data available now are not enough to establish their causality relationship. Further basic research is warranted to clarify the relationship between the kidney injury and CD.

This study has several limitations. As a single-center investigation describing baseline characteristics and renal manifestations of CD, our findings may be subject to selection bias since our institution serves as a nephrology referral center with patients predominantly from North China. Additionally, the extended study period introduced confounding factors, including mortality from pancreatic cancer and other solid tumors, as well as COVID-19 and other infectious diseases during follow-up. Renal biopsy was influenced by the admitting department, patient willingness, and whether the patient met the biopsy criteria, which may introduce some bias into the results.

## Supplementary Information

Below is the link to the electronic supplementary material.

Supplementary Material 1 (XLSX 837 KB)

## Data Availability

The datasets generated during and/or analysed during the current study are available from the corresponding author on reasonable request.
